# Outcomes of Hospitalized Patients Undergoing Endoscopic Retrograde Cholangiopancreatography (ERCP) With and Without a History of Peripheral Artery Disease

**DOI:** 10.7759/cureus.26585

**Published:** 2022-07-05

**Authors:** Ayham Khrais, Aaron Kahlam, Anmol Mittal, Sushil Ahlawat

**Affiliations:** 1 Department of Medicine, Rutgers University New Jersey Medical School, Newark, USA; 2 Department of Gastroenterology and Hepatology, Rutgers University New Jersey Medical School, Newark, USA

**Keywords:** post-procedural bleeding, advanced endoscopy, complications, peripheral artery disease, ercp

## Abstract

Introduction

Peripheral artery disease (PAD) is a common illness associated with an increased risk of complications and mortality. Gastroenterologists considering endoscopic retrograde cholangiopancreatography (ERCP) in these patients should weigh the benefits and risks carefully. Our goal is to analyze the hospital burden and complication rates in patients with PAD undergoing ERCP.

Methods

Using data from the National Inpatient Sample (NIS), patients over the age of 18 with and without PAD undergoing ERCP were identified utilizing the International Classification of Diseases (ICD)-9 codes. Primary outcomes included inpatient mortality, length of stay, and hospital charges. Secondary outcomes included rates of bile duct perforation, post-ERCP bleeding, acute pancreatitis, and cholangitis. Supplemental data, including household income and primary payer, were also analyzed. Independent t-tests were used for continuous data, chi-square tests for categorical data, and confounding variables (diabetes, age, gender, race) were controlled via multiple logistic regression.

Results

Most of the PAD group were male, while those in the non-PAD group were female (adjusted p<0.05). Mortality was higher in the PAD group (11.2% versus 8%; adjusted p<0.05). Members of the PAD group had longer lengths of stay (11.6 days versus 11 days; adjusted p*<*0.05) and more costly hospital stays ($108,006.49 versus $94,399.09; p<0.05). Members of the PAD group had higher rates of post-ERCP bleeding (5.2% versus 3.7%; adjusted p<0.05) and lower rates of cholangitis (6% versus 4%; adjusted p<0.05) and acute pancreatitis (6.9% versus 3.4%; adjusted p<0.05).

Conclusion

Patients with PAD had an increased hospital burden but had a decreased risk of post-ERCP complications, including cholangitis and pancreatitis. Physicians performing risk stratification for patients with PAD undergoing ERCP must consider these specific complications and ensure that patients undergoing this procedure are fully aware of the dangers and benefits of ERCP prior to consenting to the procedure.

## Introduction

Peripheral artery disease (PAD) describes arterial atherosclerosis leading to decreased end-organ perfusion. Between 1999 to 2000, the United States Health and Nutrition Survey found that 4.3% of individuals over the age of 40 and 14.5% of individuals over the age of 70 had PAD [1,2]. This disease is not only highly prevalent but is also associated with significant comorbidities. Of patients with PAD, 46-68% are found to have either coronary artery disease (CAD) or cerebrovascular disease [3,4]. PAD, defined as an ankle-brachial index (ABI) less than 0.9, puts patients at an increased risk for myocardial infarction, stroke, functional decline, and all-cause mortality [5-7]. This increased risk is an important decision factor for providers performing surgeries and procedures on these patients.

Despite the increased risk of mortality, and relative prevalence of the disease, there is sparse literature describing the impact of PAD on endoscopic procedures, especially endoscopic retrograde cholangiopancreatography (ERCP). ERCP can result in multiple complications, including post-ERCP pancreatitis (PEP), perforation and infection, with approximate incidences of 3.5%, 0.6%, and 1.4%, respectively [8]. Additionally, ERCPs are advanced procedures that require adequate sedation by the gastroenterologist or anesthesiologist, with additional risk for complications related to medication use and endotracheal intubation [9,10]. The risk of bleeding in patients with PAD may be higher than that of the general population, as those with PAD are usually prescribed antiplatelet medications [11,12]. Understanding the impact of these risk factors on outcomes of patients undergoing ERCP is important for the gastroenterologist when weighing the risks and benefits of performing the procedure. Since PAD is a highly prevalent disease and ERCP is associated with more complications than any other endoscopic procedure, investigating differential incidences of procedural complications among those with and without PAD would prove significantly beneficial in risk-stratifying members of this population. 

PAD is associated with other atherosclerotic syndromes, including chronic mesenteric ischemia and stenosis of other arteries that supply visceral organs [13]. We hypothesize that in patients with PAD who are predisposed to atherosclerosis, endothelial dysfunction of vessels supplying the biliary system can potentially impair post-procedural healing, thereby predisposing one to complications such as bleeding, infection, and perforation after trauma caused by ERCP.

In this study, we aim to clarify the difference in complication rates for patients with and without PAD undergoing ERCP. Specifically, we analyze the differences in mortality, length of stay, and cost, as well as complications of ERCP, such as bleeding, PEP, and infection. Furthermore, we determine differences in household income and primary insurance among both populations. This allows analysis of potential barriers to patients receiving care, including limited income and low-quality insurance. By understanding these barriers, were are able to better speculate as to variation in outcomes among members of both groups. Ultimately, we hope the results of this study clarify the increased risk that patients with PAD may carry when undergoing ERCP and aid the physician in assessing the benefits of the procedure.

## Materials and methods

Data source 

The study population consisted of patient information found in the National Inpatient Sample (NIS), the largest public all-payer inpatient database harboring information on more than seven million hospital stays in the United States. Developed by the Agency for Healthcare Research and Quality, the NIS contains no patient or hospital identifiers and provides a nationally representative set of data. Data in the NIS represents 20% of all discharges from community and academic hospitals within the United States. Sampling weight is applied annually, enabling precise national estimates. The NIS was investigated for hospitalized cases from 2001 to 2013 using the International Classification of Diseases, Ninth Revision, Clinical Modification (ICD-9 CM) codes to identify patients with PAD undergoing ERCP (see Table 1). Due to its widespread use in multiple research endeavors, as well as its precise representation of the national inpatient population, the NIS was utilized for this study. 

**Table 1:** Variables studied and respective ICD-9 codes ERCP - endoscopic retrograde cholangiopancreatography; PAD - peripheral artery disease; ICD - International Classification of Diseases

Variable	ICD-9 code
ERCP	51.10, 51.11, 51.85, 51.88, 52.13, 52.14, 52.93
PAD	443.9, 443.81, 440.20, 440.21, 440.22, 440.23, 440.23, 440.29
Post-procedural bleeding	998.1, 998.11, 998.12, 998.13
Bile duct perforation	576.3
Cholangitis	576.1
Post-ERCP pancreatitis	577.0

Study design 

This cross-sectional study utilized ICD-9 CM codes to identify all patients >18 years old undergoing ERCP. The resulting data was then stratified based on the presence of ICD-9 CM codes for PAD. In effect, two groups were created: patients undergoing ERCP with PAD and those undergoing ERCP without PAD. Primary outcomes included inpatient mortality, length of stay (LOS), and hospital charges. Secondary outcomes included possible complications of ERCP, such as cholangitis, post-procedure bleeding, acute pancreatitis, and bile duct perforation. Supplementary patient demographics (age, gender, race, median household income, and primary payer) were obtained and analyzed as well. Institutional Review Board (IRB) approval was not obtained as the NIS database is a de-identified database.

Statistical analysis 

The IBM SPSS Statistics 24 (IBM Corp., Armonk, NY, USA) software was utilized to conduct statistical analyses. Outcomes and demographic data for both tested groups were assessed via independent t-tests (for continuous data) and chi-squared tests (for categorical data). Multiple logistic regression was utilized to characterize primary and secondary outcomes among both groups while controlling for the following patient characteristics: diabetes mellitus, age, gender, and race. Statistical significance was indicated with a p-value <0.05. Adjusted odds ratios (AOR) and associated 95% confidence intervals (CI) were calculated. 

## Results

From 2001 to 2013, 961,408 patients underwent ERCP. Within that population, 33,795 patients had a diagnosis of PAD, and 927,613 did not. Patient demographics, including sex at birth, race, age, primary payer, and median household income, are found in Tables 2-4. Female patients made up the majority of the group that underwent ERCP without a diagnosis of PAD, composing 53.9% of that sample (Table 2). On the other hand, there was a greater percentage of males in the PAD group (56.1%), which consisted of only 43.9% of females (p<0.05). Patients with PAD were, on average, older (71.15 years versus 58.82; Table 3). In terms of racial distribution (Table 4), most patients in both the non-PAD and PAD groups were Caucasian, with a composition of 71.3% and 77.7%, respectively (p<0.05). In terms of median household income (Table 4), most patients in both the non-PAD (47.2%) and PAD groups (44.5%) were in the highest quartile (p<0.05). Most patients in both groups had Medicare as their primary payer; however, a significantly higher percentage of PAD patients utilized Medicare (75.9% versus 47.4%), while a significantly higher proportion of non-PAD patients utilized private insurance (32.2% versus 15.9%; p<0.05). 

**Table 2:** Differences in sex at birth ERCP - endoscopic retrograde cholangiopancreatography; PAD - peripheral artery disease; OR - odds ratio; CI - 95% confidence interval

		ERCP without PAD (n=927,613)		ERCP with PAD (n=33,795)		OR	CI	p-value
		Percentage	n	Percentage	n			
Sex at birth	Female	53.9	500,527	43.9	14,822	0.666	0.651-0.681	<0.05
	Male	46.1	426,522	56.1	18,970			

**Table 3:** Differences in age distribution ERCP - endoscopic retrograde cholangiopancreatography; PAD - peripheral artery disease; CI - 95% confidence interval; SD - standard deviation; SE - standard error

	ERCP without PAD			ERCP with PAD			Mean difference	CI	p-value
	Mean	SD	SE Mean	Mean	SD	SE Mean			
Age at admission (years)	58.82	20.647	0.021	71.15	11.410	0.062	-12.327±0.113	-12.548 to -12.105	<0.05

**Table 4:** Differences in patient demographics, including race, primary insurance payer and median household income ERCP - endoscopic retrograde cholangiopancreatography; PAD - peripheral artery disease

		ERCP without PAD		ERCP with PAD		p-value
		Percentage	n	Percentage	n	
Race	Caucasian	71.3	534,360	77.7	22,236	<0.05
	Black	10.6	79,159	10.6	3,034	
	Hispanic	12.0	89,810	7.7	2,199	
	Asian or Pacific Islander	2.5	18,819	1.3	373	
	Native American	0.6	4,485	0.4	128	
	Other	3.0	22,828	2.3	662	
Primary payer	Medicare	47.4	443,423	75.9	25,616	<0.05
	Medicaid	11.1	103,823	4.9	1,657	
	Private insurance	32.2	300,996	15.9	5,378	
	Self-pay	5.3	50,022	1.5	498	
	No charge	0.6	5,546	0.2	82	
	Other	3.4	31,764	1.5	505	
Median household income	Lowest quartile	5.2	6781	5.5	203	<0.05
	Second quartile	21.2	27,650	23.4	856	
	Third quartile	26.4	34,556	26.6	973	
	Highest quartile	47.2	61,719	44.5	1629	

Mortality, as seen in Table 5, was significantly higher in the PAD group (11.2% versus 8%; p<0.05; OR 1.433), with continued statistical significance after adjustments for confounders were made (adjusted p <0.05; AOR 1.141). Length of stay (Table 6) was greater in the PAD group (11.6 days versus 11 days), with continued statistical significance after adjusting for confounders (adjusted p<0.05; AOR 0.999). Total charges (Table 6) were also higher in the PAD group ($108,006.49 versus $94,399.09), with statistical significance that persisted despite correction for confounding variables (adjusted p<0.05). 

**Table 5:** Differences in inpatient mortality ERCP - endoscopic retrograde cholangiopancreatography; PAD - peripheral artery disease; OR - odds ratio; CI - 95% confidence interval; AOR - adjusted odds ratio; ACI - adjusted confidence interval

	ERCP without PAD (n=927,613)		ERCP with PAD (n=33,795)		OR	CI	p-value	AOR	ACI	Adjusted p-value
	Percentage	n	Percentage	n						
Mortality	8.0	75,055	11.2	3,787	1.433	1.384-1.483	<0.05	1.141	1.098-1.185	<0.05

**Table 6:** Differences in length of stay and total hospital charges ERCP - endoscopic retrograde cholangiopancreatography; PAD - peripheral artery disease; CI - 95% confidence interval; AOR - adjusted odds ratio; ACI - adjusted confidence interval; SD - standard deviation; SE - standard error; LOS - length of stay

	ERCP without PAD			ERCP with PAD			Mean difference	CI	p-value	AOR	ACI	Adjusted p-value
	Mean	SD	SE Mean	Mean	SD	SE Mean						
LOS (days)	11	14.355	0.015	11.6	12.278	143.441	-0.585±0.079	-0.74 to -0.43	<0.05	0.999	0.998-1	<0.05
Total charges (USD)	94,399.09	136,913.615	143.441	108,006.49	124,364.054	683.618	-13,607.405±763.797	-15,104.422 to -12,110.388	<0.05	1	1-1	<0.05

In terms of ERCP complications (Table 7), rates of post-ERCP bleeding were higher in the PAD group (5.2% versus 3.7%; p<0.05; OR 1.432), despite correction for diabetes mellitus, age, sex at birth, and gender (adjusted p<0.05; AOR 1.314). On the other hand, rates of bile duct perforation (0.03% versus 0.01%; adjusted p=0.061; AOR 0.264), cholangitis (6% versus 4%; adjusted p<0.05; AOR 0.534), and post-ERCP pancreatitis (PEP; 6.9% versus 3.4%; adjusted p<0.05; AOR 0.552) were higher in the non-PAD group. Rates of bile duct perforation were statistically insignificant before and after adjustment for confounders. Rates of all other studied complications were statistically significant before and after adjustment. 

**Table 7:** Rates of ERCP complications between patients with and without PAD ERCP - endoscopic retrograde cholangiopancreatography; PAD - peripheral artery disease; OR - odds ratio; CI - 95% confidence interval; AOR - adjusted odds ratio; ACI - adjusted confidence interval

	ERCP without PAD		ERCP with PAD		OR	CI	p-value	AOR	ACI	Adjusted p-value
	Percentage	n	Percentage	n						
Bile duct perforation	0.03	280	0.01	4	0.392	0.146-1.052	0.054	0.264	0.065-1.065	0.061
Cholangitis	6.0	55,915	4.0	1,369	0.658	0.623-0.695	<0.05	0.534	0.503-0.566	<0.05
Post-ERCP bleeding	3.7	34,253	5.2	1,759	1.432	1.363-1.504	<0.05	1.314	1.245-1.387	<0.05
Post-ERCP pancreatitis	6.9	63,725	3.4	1,159	0.481	0.454-0.511	<0.05	0.552	0.517-0.588	<0.05

## Discussion

PAD is a highly prevalent disease, especially in older adults, that not only affects the lower extremities but also signifies widespread cardiovascular disease. These patients are at increased risk of cardiovascular disease, cerebrovascular disease, and overall mortality and thus require important considerations when undergoing invasive procedures [5-7]. Our results show that patients with PAD are at increased risk of mortality following ERCP, have a LOS almost one day longer, have a higher cost for their overall hospitalization, and are at an increased risk of bleeding. Interestingly, we find these patients are at a lower risk of bile duct perforation, cholangitis, and PEP than their non-PAD counterparts.

While periprocedural outcomes in patients with cardiovascular disease have been well studied [14-16], there is little literature on outcomes in patients with PAD undergoing upper or lower endoscopy. Patients with PAD undergoing transcatheter aortic valve repair have been found to have an increased length of stay and increased overall cost [17]. We similarly found an increased length of stay and cost in patients with PAD undergoing ERCP, likely a result of patients with PAD having other risk factors, including smoking history, hyperlipidemia, and diabetes complicating their hospital stay [18,19]. Additionally, the presence of PAD likely indicates the presence of coronary artery disease, which may explain the increase in mortality found in our study [5,20]. Patients with PAD are already at higher risk of death from their disease, and undergoing a complex procedure such as ERCP would reasonably increase that risk compared to patients without PAD. These results highlight the importance of the risk/benefit discussion with patients who may need an ERCP.

The increased risk of bleeding observed in this study may be related to antiplatelet and anticoagulation medications that patients take for their PAD. Recent studies in PAD recommend that patients take aspirin with an oral anticoagulant, such as rivaroxaban, to improve outcomes [21-23]. Antiplatelet agents taken within six days of ERCP have been shown to increase the risk of bleeding following sphincterotomy [24]. Therefore, this patient population is prone to post-procedural bleeding due to these medications. The risks and benefits of continuing this therapy must be discussed with the patient, especially when temporarily held for impending procedures such as ERCP. It is important for gastroenterologists to not only counsel patients effectively but also collaborate with other specialties to ensure the procedure can be done safely.

Interestingly, we found that patients with PAD had a lower risk of PEP and cholangitis. Since many risk factors for PAD, including tobacco use and diabetes, increase the risk for pancreatitis, this finding was perplexing. Though controversial, some literature has shown that rectal nonsteroidal anti-inflammatory drugs (NSAIDs) may reduce PEP in patients undergoing ERCP [25-27]. Many PAD patients are on a drug regimen that includes aspirin, as discussed above. Thus, it is possible that the anti-inflammatory properties of aspirin may be suppressing the inflammatory response of the body. While no other studies have been done looking at PAD and PEP, some studied ERCP outcomes in patients with a history of percutaneous intervention (PCI) or coronary artery bypass grafting (CABG) and found that they also had a lower rate of PEP [28]. Since the risk factors for PAD are similar to those of patients with CAD requiring PCI or CABG, lower rates of PEP in the latter can be attributed to either shared comorbidities among both groups or, more likely, to the anti-inflammatory medications that patients from both groups are likely to be prescribed. Furthermore, studies involving the anti-bacterial capability of aspirin have shown that the salicylate medication has anti-staphylococcal properties via inhibition of two key virulence genes [29], as well as the capacity to degrade bacterial biofilm in vitro [30]. Specifically, aspirin-enabled degradation of biofilm produced by common causative agents of cholangitis (including gram-negative organisms) could explain the lower incidence following ERCP in patients with PAD. While it seems unlikely that vascular disease would provide a protective effect on PEP, further studies looking at the impact of aspirin and other anti-inflammatory medications are needed. Despite the decreased risk in ERCP-related adverse outcomes, LOS was still longer in these patients, suggesting that other factors, such as comorbidities, advanced disease, and increased risk of mortality, may play an important role. The significantly larger percentage of Medicare as a primary payor in the PAD group highlights the economic disparity between patients with multiple comorbidities that place them at risk for PAD and those that do not. Patients with lower income would be unable to afford to exercise and purchase healthy food, thus placing them at higher risk for hypertension and diabetes, thereby increasing the prevalence of PAD in this population [1,2]. 

There are some important limitations to this study. The NIS database relies on correct ICD diagnosis and procedure codes to be entered, and errors in coding may result in inaccurate data. Furthermore, not all patients with PAD may have had an associated ICD diagnosis code for PAD entered during hospitalization for ERCP. Additionally, data from the NIS only reflects one hospitalization and cannot be used for longer-term outcomes. Furthermore, details about medication and treatment plans are not available outside of ICD codes, making it difficult to assess the effects of different therapies. 

## Conclusions

Hospital burden, including LOS, cost, and overall mortality, was higher in patients with PAD undergoing ERCP. Additionally, bleeding risk was higher in these patients, likely due to the antiplatelet and antithrombotic treatment regimens the patients are on. Interestingly, PEP and cholangitis rates after ERCP were lower in the PAD group. Further research into these surprising results may clarify ways in which physicians can reduce risks associated with ERCP and improve overall outcomes.
